# Tuberculous liver abscess as a unique cause of liver abscess: A case report and literature review

**DOI:** 10.1016/j.heliyon.2023.e20755

**Published:** 2023-10-06

**Authors:** Elabbass A. Abdelmahmuod, Mahmoud Elayana, Eihab Subahi, Loai Aker, Mohammed A. Alamin, Gamal Alfitori

**Affiliations:** a Department of Internal Medicine, Hamad Medical Corporation, Doha, Qatar; b Department of Clinical Imaging, Hamad Medical Corporation, Doha, Qatar

**Keywords:** Tuberculosis, TB, Liver abscess, Tuberculous abscess, Immunocompetent

## Abstract

**Introduction:**

TLA is most commonly associated with an immunocompromised state, a focus of infection in the lungs or gastrointestinal system, or as part of congenital or miliary tuberculosis. Isolated TLA is rare, with only a few cases reported in the literature.

**Methods:**

We describe a case of a 24-years-old healthy male with an isolated Tuberculous Liver abscess presented with prolonged fever, abdominal pain, and general malaise. He was successfully treated with a 6-month antituberculosis regimen and percutaneous abscess drainage.

**Discussion and conclusion:**

The signs and symptoms of isolated TLA are nonspecific. The diagnosis requires a high index of suspicion, especially in endemic areas and in individuals with a known tuberculosis risk factor. A better outcome is linked to an early diagnosis and timely treatment with systemic Antituberculous medications. This case report highlights the importance of considering TLA (Tuberculous or Tubercular Liver Abscess) when diagnosing hepatic masses or abscesses as a possible cause of extrapulmonary tuberculosis (EPTB).

## 1 Introduction

Hepatic tuberculosis (TB) is frequently associated with pulmonary tuberculosis (TB) or tuberculous enterocolitis (TEC). It is rare to get an isolated tuberculous liver abscess. Very few reported cases in the literature described this rare clinical entity [1].

Hepatic tuberculoma is characterized by the formation of granulomas, which may heal with localized fibrosis and calcification or merge to create tuberculomas or necrosis, which can lead to an abscess if the lesion is large enough [2].

Because pulmonary tuberculosis is becoming more common, Physicians should be mindful of the risk of tuberculous infection in all patients with non-resolving liver abscesses and unknown hepatic mass lesions, especially in areas with high TB prevalence.

The three morphologic types of liver TB include focal/local tuberculoma or abscess, diffuse liver infiltration without apparent pulmonary involvement, and diffuse liver involvement with pulmonary or miliary tuberculosis [13].

## 2 Case report

A 24-year-old male with no past medical history of note presented with two weeks history of intermittent fever and generalized fatigability. Three days before admission, he developed abdominal pain that started as mild in the epigastrium, then became more severe and spread to the rest of the abdomen. It was associated with nausea and a few episodes of vomiting with no change in his bowel habit. On admission, he was febrile with a temperature of 38.5 °C. The general and systemic examinations, apart from a generalized abdominal tenderness, were unremarkable. His initial labs showed raised white blood cells and high C reactive protein (see Table 1, Table 2). Liver function test and Chest x-ray (CXR) were normal.

**Table 1:** CBC and inflammatory markers.

Detail	Value w/Units	Normal Range
Lactic Acid	1.7 mmol/L	0.5–2.2
WBC	20.7 x10^3/uL	4.0–10.0
Hgb	12.7 gm/dL	13.0–17.0
RDW-CV	13.7%	11.6–14.5
CRP	109 mg/L	0–5

**Table 2:** Renal and liver functions tests.

Detail	Value w/Units	Normal Range
Urea	5.3 mmol/L	2.5–7.8
Creatinine	99 μmol/L	64–110
Sodium	136 mmol/L	133–146
Potassium	4.0 mmol/L	3.5–5.3
Chloride	98.7 mmol/L	95.0–108.0
Bicarbonate	22.0 mmol/L	22.0–29.0
Bilirubin T	12.1 μmol/L	3.4–20.5
Total Protein	78 gm/L	60–80
Albumin Lvl	41 gm/L	35–50
Alk Phos	118 U/L	40–150
ALT	11 U/L	0–55
AST	24 U/L	5–34

A computerized tomographic (CT) scan of the abdomen showed diffuse abdominal lymphadenopathy and a left hepatic lesion in the segment at a subcapsular region which appeared inseparable from an enlarged lymph node [Fig. 1(a and b)]. An Ultrasound abdomen confirmed the diagnosis of the left hepatic abscess (5.2 × 2.9cm with a volume of 26 cc) [Fig. 2 (a,b)].

**Image 1:**
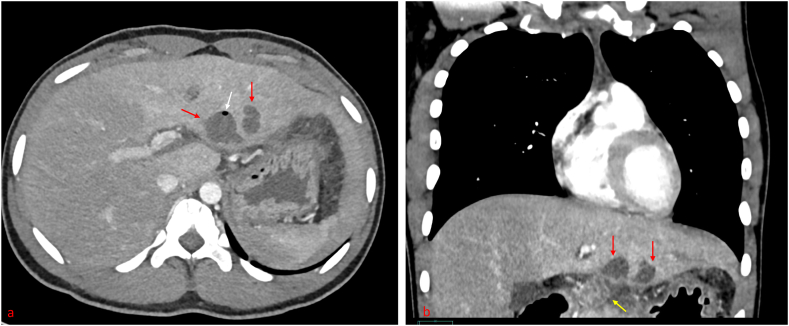
CT abdomen reflected small liver abscess.

**Image 2:**
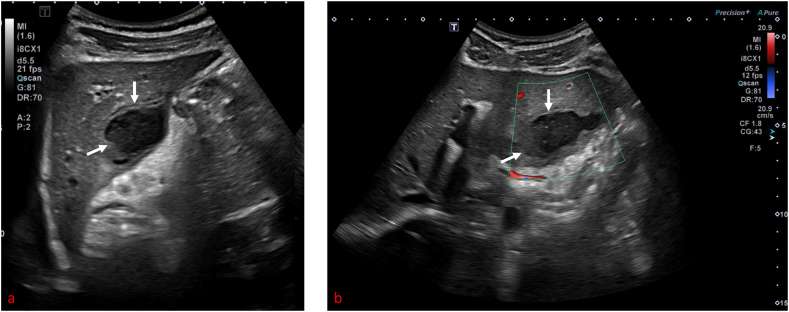
US abdomen confirmed the diagnosis.

Routine and microscopic examinations of stool revealed no cyst or ova, and the HIV test was negative, as well as the amoebic serology.

Liver abscess drainage was performed under US guidance, and the sample was sent for microbiological investigations, including gram stain, culture, and TB workup. All were negative except for the positive TB PCR; hence the TLA diagnosis was made. He was started and improved on Anti-TB medications. At the regular follow-ups in the TB clinic, he remained well and asymptomatic.

## 3 Discussion

Hepatic tuberculosis has been classified as a rare but not exceptional type of extrapulmonary tuberculosis. A tuberculous liver abscess (TLA) is, on the other hand, a highly uncommon [3]. Most cases are associated with miliary pulmonary tuberculosis and mainly spread through hematogenous dissemination [4]. In our case, there was no evidence of tuberculous involvement in the patient's lungs or the gastrointestinal tract.

Bestow published the first description of TLA in 1858. TLA was found to be present in only 0.34% of patients with hepatic TB, according to a study in which patients ranged in age from 6 months to 72 years, with an average age of 39.2 years [1]. The diagnosis of TLA is difficult and usually made at autopsy or sometimes if a laparotomy has been done [5].

In endemic regions, hepatic TB can present in the form of a hepatic abscess, as an intrahepatic mass, or as granulomatous hepatitis (fever with hepatomegaly and mildly deranged liver function tests) [6]. The presentation of the TLA is usually nonspecific symptoms such as fever, vague abdominal pain, malaise, and weight loss. Hepatomegaly is a common finding on examination, but jaundice is rarely encountered and usually suggests biliary involvement in the form of extra or intrahepatic obstruction. There is no apparent link between the severity of liver disease and jaundice [7]. Isolated TLA is a very rare condition but should always be considered when a patient has a liver abscess, especially if from an endemic region.

One should be suspicious of isolated tuberculous abscesses if there is a history of fever and liver abscess. Diagnosis depends on imaging studies such as an abdominal USG or CT scan, but the diagnosis is more certain when tubercle bacilli are isolated from a pus aspirate [14].

US and CT scans have limited specificity for TLA. However, they are instrumental in determining the abscess' location, size, and multiseptated nature. Their findings typically indicate various stages of the disease, ranging from granulomatous tubercles with or without caseous necrosis through fibrosis and calcification in the healing stage [8]. The presence of acid-fast bacilli (AFB) in the aspirated pus, a pus culture indicating Mycobacterium Tuberculosis, or a positive ELISA and PCR for Mycobacterium Tuberculosis are required to confirm the diagnosis [9].

AFB is most easily discovered in a caseous necrotic tissue; however, its absence should not rule out the diagnosis completely, especially in a region with a high TB incidence rate, as some previous studies have shown. The PCR technique has recently been shown to be very helpful in diagnosing liver TB [10].

• The Xpert MTB/RIF assay (Cepheid, Sunnyvale, CA, USA) has been successfully used to diagnose lymph node and pulmonary tuberculosis. Its efficacy in some types of additional extrapulmonary TB has also been studied. It is not known how Xpert MTB/RIF is used in the diagnosis of tuberculous liver abscess [15].

A six month of quadruple antituberculous therapy is indicated, and it was successfully advocated in our patient. Some support that the solid fibrous tissue surrounding the abscesses and their vast size may prevent the antibiotics from reaching the field [11]. In some cases, TLA has been successfully treated by percutaneous drainage combined with antitubercular medication transcatheter infusion. Surgery is reserved for cases where percutaneous aspiration fails or in complicated abscesses with a multiseptated field [12].

## 4 Conclusion

Only a few isolated Tuberculous Liver Abscess (TLA) cases without active pulmonary or miliary tuberculosis or other clinical signs of tuberculosis have been reported in the literature.

The symptoms and signs of isolated TLA are non–specific, and the diagnosis requires a high index of suspicion, especially in endemic TB areas and in individuals with known tuberculosis risk factors. A favorable outcome is linked to an early diagnosis and timely treatment with systemic Antituberculous medications.

## Statement of ethics

This case was approved by the Hamad Medical Corporation's Medical Research Center by the number MRC-04-21-1001.

Written informed consent was obtained from the patient to publish this case report.

## Funding sources

None.

## Data availability statement

Data and materials are available on reasonable request.

## CRediT authorship contribution statement

**Elabbass A. Abdelmahmuod:** Conceptualization, Data curation, Writing – original draft, Writing – review & editing. **Mahmoud Elayana:** Writing – review & editing. **Eihab Subahi:** Writing – review & editing. **Loai Aker:** Data curation, Investigation, Resources, Software. **Mohammed A. Alamin:** Writing – review & editing. **Gamal Alfitori:** Conceptualization, Supervision, Writing – review & editing.

## Declaration of competing interest

The authors declare that they have no known competing financial interests or personal relationships that could have appeared to influence the work reported in this paper.
